# Toward AI-Driven Detection of Asymptomatic Chronic Conditions from Stool Metagenomics and Dietary Data: A Multimodal Deep Learning Framework for T1DM, T2DM, MOS/PCOS, Cancer, and Autoimmune Disease

**DOI:** 10.3390/microorganisms14091880

**Published:** 2026-08-24

**Authors:** Károly Szili, Csilla Dézsi, Viktor Gulyás-Oldal, Dániel Sallai, Gábor Patay, Ekaterine Paschali, Sándor Nagy

**Affiliations:** 1 Department of Obstetrics and Gynecology, Faculty of Health and Sport Sciences Széchenyi István University of Győr, Egyetem tér 1, 9026 Győr, Hungarygoviktor93@gmail.com (V.G.-O.); sallai.daniel@yahoo.com (D.S.); patay@microbiomebank.com (G.P.); drpaschali@hotmail.com (E.P.); 2 Department of Obstetrics and Gynecology, Faculty of Medicine, University of Szeged, 6720 Szeged, Hungary

**Keywords:** gut microbiome, machine learning, type 1 diabetes mellitus, type 2 diabetes mellitus, metabolic obesity syndrome, polycystic ovary syndrome, colorectal cancer, autoimmune disease, precision nutrition, explainable AI

## Abstract

Chronic non-communicable conditions—type 1 and type 2 diabetes mellitus (T1DM, T2DM), metabolic obesity syndrome (MOS), polycystic ovary syndrome (PCOS), colorectal and extra-intestinal cancers, and systemic autoimmune disease—share a prolonged asymptomatic phase during which conventional screening is invasive, insensitive, or resource-intensive. This review synthesizes the 2021–2026 literature on fecal microbiome-based artificial intelligence (AI) diagnostics across these conditions, extracting reported discrimination, validation strategy, microbial and short-chain fatty acid (SCFA) biomarkers, and cross-cohort reproducibility. Across the primary classifier studies tabulated here, reported areas under the curve (AUCs) span 0.76–0.99 under internal validation but 0.69–0.91 under external or cross-population validation; in the four studies reporting both, the median AUC falls from 0.875 to 0.810. Verified external-validation values include 0.82 for colorectal cancer, 0.79 for T2DM and 0.792 for discrimination of systemic lupus erythematosus from rheumatoid arthritis and controls. Clinical readiness turns on this internal-to-external gap more than on the headline AUC. We propose a multimodal deep learning architecture coupled with explainable AI; no component has been implemented or evaluated on data, and it is presented as a design proposal. Fecal-microbiome-based multimodal AI is technically feasible but clinically unvalidated, pending prospective, harmonized cross-cohort trials.

## 1. Introduction

Chronic non-communicable diseases—including type 1 diabetes mellitus (T1DM), metabolic obesity syndrome (MOS), colorectal cancer (CRC), and systemic autoimmune disorders—collectively account for the majority of global disease burden, yet frequently remain undetected until clinically overt, at which point curative opportunities are substantially reduced [1]. The asymptomatic phase of these conditions can extend for years, representing a critical but underutilized window for intervention. Conventional screening strategies—HbA1c testing, colonoscopy, autoantibody panels—are either invasive, low-sensitivity in early stages, or resource-intensive for population-scale deployment [2].

The human gut microbiome functions as a dynamic interface between host metabolism, immunity, and the environment [3]. Dysbiosis—disruption of normal microbial community structure and function—has been implicated in the pathogenesis of T1DM, MOS, CRC and autoimmune disease, making fecal microbiome profiling an attractive non-invasive biomarker source; non-invasive, microbiome-based diagnosis has already been demonstrated at scale in inflammatory bowel disease [4]. Stool samples are easily obtainable, stable under standardized collection protocols, and yield richly informative metagenomic data through both 16S rRNA amplicon sequencing and shotgun whole-genome metagenomics.

Over 2021–2026, artificial intelligence (AI) methods—particularly random forests, gradient boosting, graph neural networks, and transformer-based architectures—have demonstrated the ability to identify disease-specific microbial signatures with diagnostic accuracy that in several validation cohorts matches or exceeds conventional clinical markers [5,6]. Simultaneously, precision nutrition research has shown that dietary pattern data, when integrated with microbiome profiles, improves predictive performance for metabolic and inflammatory outcomes [7].

This review synthesizes the 2021–2026 literature on AI-driven microbiome diagnostics across four chronic disease domains, proposes a multimodal deep learning architecture for a candidate detection framework, and outlines the role of dietary data integration.

## 2. Materials and Methods

### 2.1. Search Strategy

This is a narrative review and does not claim compliance with PRISMA reporting standards; we nonetheless report the search process in full so that readers can judge the scope of the literature considered. Literature was identified between 5 and 14 July 2026 through 31 structured searches combining disease terms (type 1 and type 2 diabetes mellitus, metabolic obesity syndrome, polycystic ovary syndrome, colorectal and extra-intestinal cancer, rheumatoid arthritis, systemic lupus erythematosus, inflammatory bowel disease, psoriasis, rosacea, endometriosis) with the methodological terms “gut microbiome”, “machine learning”, “artificial intelligence”, “deep learning”, “fecal biomarker” and “short-chain fatty acid”. Searches were conducted using a web-based literature search interface that surfaces records from PubMed/PMC and publisher platforms; formal Boolean queries against MEDLINE, Web of Science or Scopus were not executed, and indexing status was applied as an eligibility criterion, not as a search filter. These 31 searches returned 252 result items—individual records or record clusters returned by the interface. Thirteen further searches were run over the same interface and in the same period to identify a suitable target journal, returning 119 result items; they are listed in Table S1 for completeness, form no part of the evidence base, and no record was retained from them. Deduplication was carried out across the complete July retrieval log—371 result items in total—and yielded 331 unique records. Cited sources were drawn from the topic-search subset only; no record retrieved solely by a journal-selection search is cited. A further 23 targeted searches were run in August 2026 against PubMed using explicit Boolean queries to verify specific quantitative claims, to resolve head-to-head comparisons requested during peer review, and to confirm the bibliographic details of cited works. Because the retrieval described above is not reproducible by a reader, a further 13 explicit Boolean queries covering each disease domain, external validation, calibration reporting, dietary integration and prediagnostic detection were executed against PubMed through the NCBI E-utilities interface on 10 August 2026, returning 7621 records; these are reported with their exact query strings and per-query counts so that retrieval can be repeated exactly. Fourteen of the fifteen studies in Table 1 are retrieved by at least one of these confirmatory queries; the exception falls outside the 2021–2026 date limit and is cited under the primary-source attribution rule described in Section 2.2. The complete query list is provided in the Supplementary Materials (Table S1).

### 2.2. Eligibility Criteria

Two distinct criteria were applied, one governing which literature was surveyed and one governing which source is cited for a given claim. Survey scope: records were retained if they (i) were published in a peer-reviewed journal indexed in PubMed/MEDLINE or Scopus, (ii) appeared between 2021 and 2026, and (iii) reported original data, a systematic synthesis, or a methodological benchmark relevant to microbiome-based classification of the disease domains considered here. Preprints, conference abstracts and non-indexed sources were excluded from the evidence base; a clinical-trial registry entry is cited once, solely to document an ongoing trial, and is not treated as evidence. The four-year window reflects the pace at which classifier architectures and reference databases in this field are superseded; it is a criterion for representativeness of the survey, not a judgement on the validity of older work. Attribution of quantitative findings: where a specific performance figure is quoted, it is attributed to the study that originally reported it, irrespective of that study’s publication date. Secondary sources within the survey window frequently restate performance figures drawn from earlier primary work; citing the recent secondary source would satisfy the date criterion while obscuring the provenance of the number and inflating the apparent recency of the evidence base. Every figure in Table 1 is therefore cited to its primary source, with the publication year of that source given in the table.

### 2.3. Data Extraction and Synthesis

For each retained study we extracted the disease domain, cohort size, sequencing approach, classifier architecture, validation strategy, and reported discrimination (AUC), together with the principal limitation stated by the authors or evident from the design; these are tabulated in Table 1. Extraction was performed by one author and checked against the source records by a second. Findings were synthesized qualitatively. Risk of bias and applicability were appraised independently by two authors using a PROBAST-informed framework across the four PROBAST domains (participants, predictors, outcome and analysis); any difference between the two sets of judgements was to be resolved by consensus discussion, and the two assessments agreed on every appraised study. The study-level results are reported in Table S2. No formal meta-analytic pooling was attempted, because the included studies differ substantially in cohort composition, sequencing platform, taxonomic reference database, batch-correction approach and classifier architecture—heterogeneity that a pooled effect estimate would obscure rather than resolve. Risk of bias and applicability were appraised using a PROBAST-informed framework across the four PROBAST domains, based on the open-access full texts of 14 of the 15 studies in Table 1; one study was not available in open access and was not appraised. The appraisal is reported in Table S2.

### 2.4. Use of Generative Artificial Intelligence

**Figure 1:**
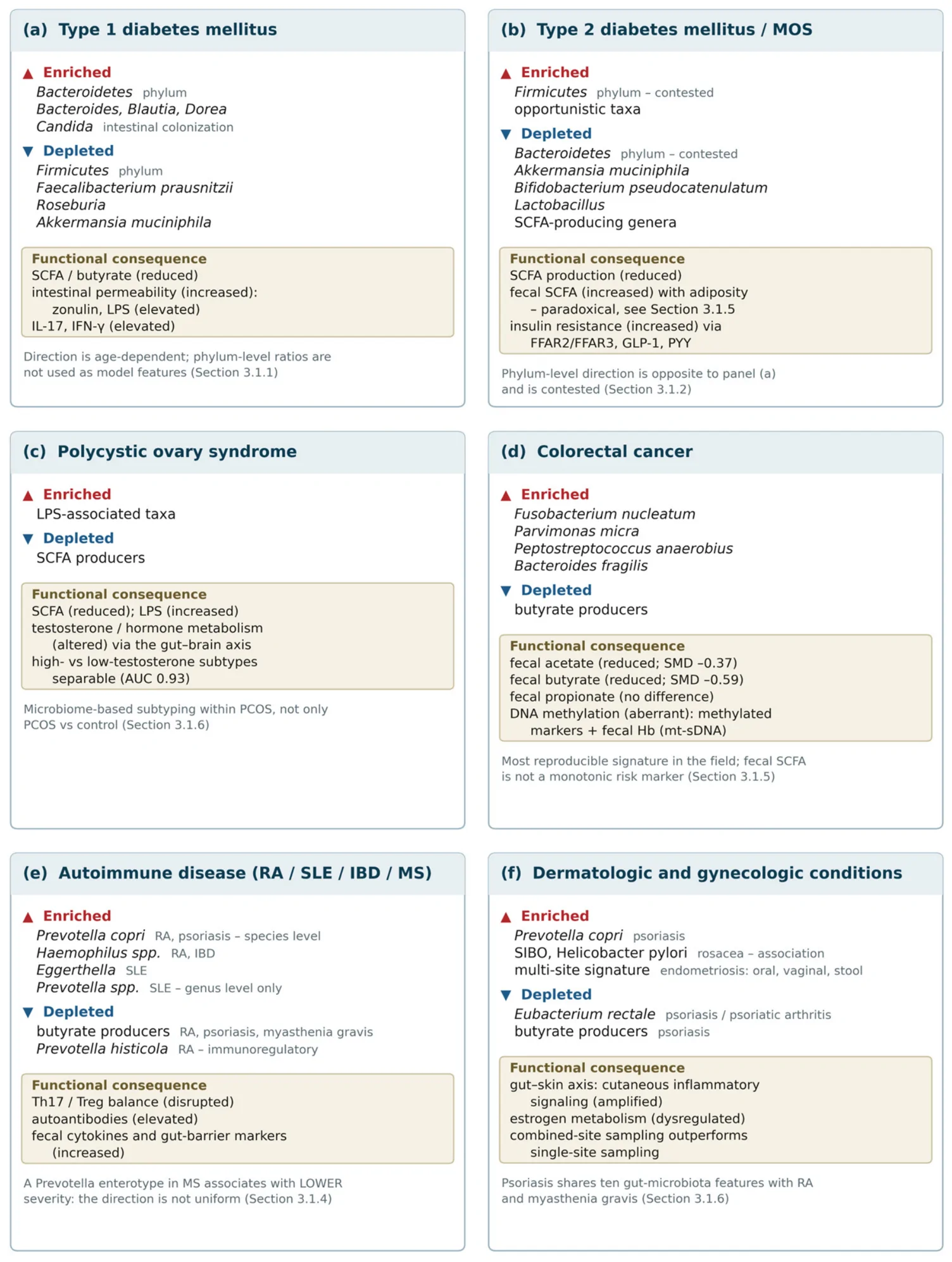
Disease-specific gut microbial marker signatures across six condition groups. For each group the enriched (▲) and depleted (▼) taxa reported in the literature reviewed are listed, together with the functional consequence written directly into the panel. T1DM (**a**) and T2DM/MOS (**b**) are shown separately because the reported phylum-level direction is opposite between them and is contested in obesity (Section 3.1.1 and Section 3.1.2); (**c**) polycystic ovary syndrome (Section 3.1.6); (**d**) colorectal cancer (Section 3.1.5); (**e**) autoimmune disease, including the multiple sclerosis counterexample in which a *Prevotella enterotype* associates with lower severity (Section 3.1.4); (**f**) dermatologic and gynecologic conditions (Section 3.1.6). Entries correspond to Table 2, where the consistency of each reported direction across independent studies is assessed.

**Figure 2:**
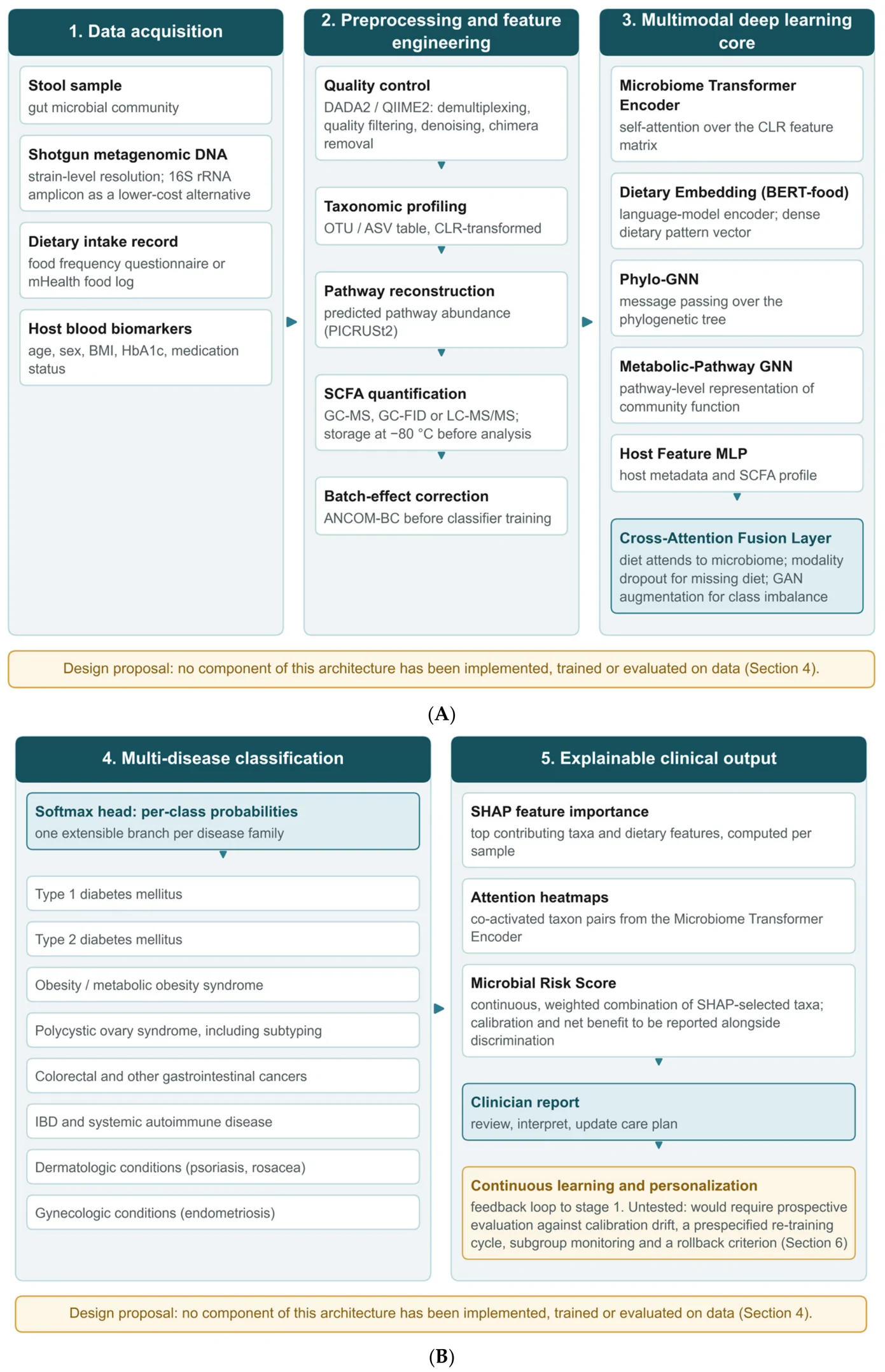
(**A**). Proposed multimodal deep learning framework, stages 1–3: data acquisition, preprocessing and feature engineering, and the multimodal deep learning core. Stool microbiome, shotgun metagenomic DNA, dietary intake records and host blood biomarkers are quality-controlled and reduced to an OTU/ASV table with reconstructed metabolic pathways and quantified SCFA, then encoded by the Microbiome Transformer Encoder, the Dietary Embedding (BERT-food), the Phylo-GNN, the Metabolic-Pathway GNN and the Host Feature MLP, combined through the Cross-Attention Fusion Layer trained with modality dropout. Stages 4–5 are shown in (**B**). No component has been implemented, trained or evaluated on data. (**B**). Proposed multimodal deep learning framework, stages 4–5: multidisease classification and explainable clinical output. A softmax head produces per-class probabilities across T1DM, T2DM, obesity/MOS, PCOS including subtyping, colorectal and other gastrointestinal cancers, inflammatory bowel disease and systemic autoimmune disease, dermatologic and gynecologic conditions. Explainability comprises SHAP feature importance, attention heatmaps and a continuous Microbial Risk Score, feeding a clinician report and a continuous learning and personalization loop back to stage 1. No illustrative risk value is shown, because the model has not been fitted; the re-training loop is a design element whose behavior is untested (Section 4 and Section 6).

As the authors are not native English speakers, a generative artificial intelligence (GenAI, Claude Opus 4.8) tool was used during manuscript preparation for language editing (grammar, spelling, and fluency improvement) and, in the originally submitted version, for figure/image editing assistance. All figures in this revised version (Figure 1, Figure 2A,B and Figure S1) have been redrawn as vector schematics generated deterministically from a plotting script and contain no AI-generated or AI-edited imagery. GenAI was not used to generate scientific content, data, or conclusions. The authors have reviewed and edited all GenAI-assisted output and take full responsibility for the content of this publication.

## 3. Results

### 3.1. Disease-Specific Microbiome Findings and Machine Learning Evidence

Reported classifier performance across the reviewed disease domains is summarized in Table 1, which lists for each study the cohort size, sequencing approach, model class, validation strategy, reported discrimination and principal limitation. We present them together because the headline AUC of a microbiome classifier means little without knowing how it was validated. Three patterns emerge, and each qualifies the optimistic reading that individual studies invite (Figure 1).

First, discrimination falls substantially under external validation. A random forest classifier for colorectal cancer achieved AUC 0.90 (95% CI 0.869–0.931) internally and 0.82 in cross-population validation [14]. A multiclass fecal classifier obtained AUROC 0.90–0.99 on an independent test set from the same population but 0.69–0.91 when applied to public datasets from other populations [21]. A systematic review of AI/ML for microbiome-based CRC screening reports internal-validation AUCs of 0.61–0.98 against external-validation AUCs of 0.70–0.87 [22]. A classifier built on microbial single-nucleotide variants retained AUCs of 0.83, 0.82 and 0.76 across three independent validation cohorts, although its sensitivity and specificity varied widely between them [17]. A cross-cohort evaluation across 20 diseases reported intra-cohort discrimination of approximately 0.77 AUC but cross-cohort discrimination of approximately 0.73 only for intestinal diseases, with non-intestinal classifiers failing to transfer [20]. Across the four studies in Table 1 reporting both an internal and an external estimate, the median AUC falls from 0.875 to 0.810 (median change −0.060; range −0.145 to +0.010); the four pairs and the convention used for reported ranges are given in the footnote to Table 1. We report this descriptively and do not pool it: with four paired observations and substantial methodological heterogeneity, no inferential test is warranted, and a meta-analytic estimate would convey a precision the evidence does not support. The range is more informative than the median. The single exception—a marginal increase of +0.010—occurred in the one study that systematically optimized the analytical pipeline before external testing, benchmarking 6468 pipeline configurations under leave-one-dataset-out validation with explicit batch correction [15]. This single observation is consistent with the generalization gap being largely a function of analytical standardization and not an intrinsic ceiling on the biological signal, but one study cannot establish it. This does not license optimism about clinical readiness: no study in Table 1 evaluated a classifier prospectively in an asymptomatic cohort, which remains the decisive untested question and is taken up in Section 5.2.

Second, sequencing depth matters, but less than the analytical pipeline. The same benchmark compared 16S rRNA amplicon and shotgun metagenomic data head-to-head on 4217 fecal samples [15]. Shotgun data outperformed 16S for both colorectal cancer (AUC 0.85 vs. 0.82 for the optimized pipeline; 0.86 vs. 0.77 averaged across pipelines) and adenoma detection (0.73 vs. 0.67; 0.64 vs. 0.61). The margin attributable to sequencing technology (+0.03 when both pipelines are optimized) is smaller than the margin attributable to pipeline choice within a single technology (+0.09 between the average and the optimized 16S pipeline). This supports the hybrid workflow proposed in Section 4.1 and indicates that standardization of the analytical pipeline is at least as consequential for clinical deployment as the choice of sequencing platform, an argument developed in Section 5.2.

Third, adding functional readouts improves discrimination measurably, whereas the incremental contribution of dietary data reported to date is small. Integrating metabolite profiles with taxonomic data raised performance from AUC above 0.70 to 0.818 in a multiomic analysis of overweight status [12], and a combined panel of five microbial and five metabolite markers distinguished colorectal adenoma from carcinoma with AUC 0.85, exceeding the specificity of CEA and CA19-9 [23]. By contrast, where the incremental value of dietary information has been quantified directly, the effect was statistically significant but numerically small (AUC 0.743 vs. 0.741, *p* = 0.013) [24]. We return to this in Section 5.1. Taken together, Table 1 supports the conclusion that microbiome-based classification is reproducible enough to justify prospective evaluation and not yet reproducible enough to justify deployment. 

#### 3.1.1. Type 1 Diabetes Mellitus (T1DM)

T1DM is an autoimmune condition driven by T-cell-mediated destruction of pancreatic beta-cells, arising from genetic susceptibility interacting with environmental triggers. Gut dysbiosis in T1DM is associated with increased intestinal permeability (“leaky gut”), allowing luminal antigens to cross the epithelial barrier and trigger or amplify autoimmune attack on pancreatic tissue [25]. This process is accompanied by elevated pro-inflammatory cytokines including IL-17 and IFN-gamma [25].

Compositionally, most published series report an increased relative abundance of Bacteroidetes and a decrease in Firmicutes in T1DM—that is, a reduced Firmicutes-to-Bacteroidetes ratio—with depletion of butyrate-producing genera and enrichment of pro-inflammatory taxa [25]. This direction is supported by primary case–control data in children [26] and by a two-sample bidirectional Mendelian randomization analysis in which the Bacteroidetes phylum showed a causal association with T1D risk (OR 1.24, 95% CI 1.01–1.53) [27]. The phylum-level signal is nonetheless age-dependent and should not be treated as a diagnostic marker: a systematic review restricted to adults with T1DM reported a predominance of Firmicutes and no significant difference in Bacteroidetes abundance [28], and the source cited above explicitly notes that despite agreement on direction there is no consensus on specific or typical biomarkers [25]. More broadly, the universal biomarker status of the Firmicutes/Bacteroidetes ratio in metabolic disease has been contested [29]. We therefore regard phylum-level ratios as unsuitable model features and use genus- and species-level abundances in the architecture proposed in Section 4. Supervised machine learning applied to 16S rRNA hypervariable features distinguished diabetes status with a mean AUC of 0.76, although the authors report that all models lacked sensitivity (0.46, rising to 0.80 only after reduction to 500 features) [8]. That estimate was obtained on a citizen-science cohort in which diabetes status was self-reported; outcome misclassification of this kind attenuates apparent discrimination, and classifiers trained on clinically confirmed diagnoses have performed substantially better (Table 1). A multiomics ML study integrating gut microbiota, serum metabolomics, and lipidomics in a T1DM cohort identified a combined panel of bacterial taxa, metabolites, and lipid species that collectively distinguished T1DM from healthy controls, with reduced *Akkermansia muciniphila* and *Faecalibacterium prausnitzii* among the most discriminative depleted taxa [9].

#### 3.1.2. Metabolic Obesity Syndrome (MOS)

MOS—encompassing insulin resistance, visceral adiposity, dyslipidemia, and low-grade systemic inflammation—is a major driver of type 2 diabetes, non-alcoholic fatty liver disease, and cardiovascular disease. Gut dysbiosis is a consistent feature, with 16S rRNA sequencing implicating a reduction in Bacteroidetes abundance alongside a corresponding elevation in Firmicutes in obese states [30]. Machine learning frameworks applied to gut microbiome data from large obese cohorts (n = 1563) have identified *Bifidobacterium pseudocatenulatum* as a candidate therapeutic target using ML-based biomarker discovery, alongside established associations between *Akkermansia muciniphila*, *Lactobacillus*, and improved glucose and insulin parameters [11,31]. Probiotic and prebiotic modulation of these taxa, combined with omega-3 fatty acid intake, has shown protective associations against metabolic endotoxemia and insulin resistance [11].

This phylum-level characterization requires qualification. A dedicated appraisal concluded that it is difficult to associate the Firmicutes/Bacteroidetes ratio with a determined health status and specifically to regard it as a hallmark of obesity [32]; a pooled re-analysis of published cohorts found no significant association with obesity either for the ratio or for the individual phylum abundances [33]. Genus- and species-level findings—depletion of *Akkermansia muciniphila*, *Bifidobacterium pseudocatenulatum* and *Lactobacillus*—have proved more reproducible than the phylum-level ratio and are the features carried forward in Table 2.

#### 3.1.3. Colorectal and Extra-Intestinal Cancer

CRC is among the most prevalent malignancies worldwide and a leading cause of cancer mortality; because early-stage detection dramatically improves survival while conventional non-invasive tests such as the fecal immunochemical test (FIT) have limited sensitivity for early lesions, microbiome-based screening is an active research priority [34]. *Fusobacterium nucleatum* is the most extensively characterized CRC-associated organism and is consistently among the taxa that discriminate CRC from controls in stool-based classifiers and in pooled cross-cohort metagenomic analyses [14,29]. A 2025 random forest model trained on five publicly available 16S rRNA datasets spanning North American and East Asian cohorts identified 109 discriminatory microbial taxa distinguishing healthy controls, adenomas, and CRC, achieving AUC = 0.90 (95% CI: 0.869–0.931) in internal validation and AUC = 0.82 in external cross-population validation, alongside a derived Microbial Risk Score (MRS) modeled on polygenic risk score methodology that was significantly elevated in CRC across cohorts [14]. A pooled analysis of 3741 stool metagenomes across 18 cohorts established cross-stage and strain-level reproducible microbial biomarkers of CRC, reinforcing that microbial profiling generalizes across populations and disease stages [16]. Explainable AI approaches applying SHAP values to CRC microbiome data have enabled clearer separation of CRC subgroups within principal component space [22]. Beyond CRC, distinctive tissue and fecal microbial signatures—including deoxycholic-acid-related taxa—have also been identified in breast cancer, and a proposed AI-powered “digital gut twin” framework aims to integrate diet, microbiome, and host data to guide precision cancer prevention strategies more broadly [7,35].

The relationship between microbiome-based classification and existing stool-based screening deserves specific comment, because discrimination and false-positive rate are distinct quantities and only the former is routinely reported. In a reproducibility study applying a previously developed machine learning gut microbiota model at a fixed cut-off, the false-positive rate among 245 healthy individuals was 7.3%, compared with 2.4% for FIT, while true-positive rates among 81 patients with CRC were 53.1% and 86.4% respectively [18]. Combining the two tests raised detection to 91.4% (78.3% at stage 0/I) but also raised the false-positive rate to 9.4%. On the available direct evidence, therefore, adding a microbiome layer to FIT increases false positives instead of reducing them, and the trade-off runs against specificity.

#### 3.1.4. Autoimmune Diseases (RA, SLE, IBD, and Others)

Autoimmune diseases share disruption of the Th17/Treg cell balance as a common pathophysiological axis, with gut dysbiosis implicated as an upstream contributor via molecular mimicry and altered mucosal immune priming [36]. A 2025 machine learning study applied to multicohort gut microbiota data across several autoimmune conditions identified both shared and disease-specific microbial signatures: *Haemophilus* spp. were enriched in rheumatoid arthritis (RA) and inflammatory bowel disease (IBD); *Eggerthella* and *Prevotella* spp. were enriched in systemic lupus erythematosus (SLE); and butyrate-producing genera were depleted across psoriasis, RA, and myasthenia gravis [36]. In IBD specifically, random forest models applied to a large multicohort metagenomic dataset (nearly 6000 samples) discriminated IBD from healthy controls, and Crohn’s disease from ulcerative colitis, with high accuracy [4]. A separate ML-based diagnostic model using fecal microbiome analysis for Crohn’s disease and ulcerative colitis, built on ANCOM-BC-corrected abundance data and a sparse partial least squares discriminant analysis (sPLS-DA) classifier, demonstrated robust multiclass discrimination across a downsampled, balanced cohort [37]. Cross-cohort validation studies evaluating gut-microbiome-based classifiers across 20 diseases confirm that IBD-type autoimmune conditions rank among the most reproducibly classifiable across independent populations [20]; the wider prospect of microbiome biomarkers across autoimmune disease has been reviewed recently [38].

The taxonomic level at which *Prevotella* is reported is consequential and is often collapsed across diseases. Antibody reactivity to *Prevotella copri* is elevated in anti-cyclic-citrullinated-peptide-positive individuals at risk of rheumatoid arthritis before clinical onset [39], and species-level expansion of *P. copri* is reported in psoriasis [40]. In systemic lupus erythematosus the reported enrichment is at the genus level (*Prevotella* spp.) [36]; we are not aware of species-resolved evidence establishing *P. copri* specifically in SLE. The genus is moreover not directionally uniform across autoimmune disease: *Prevotella histicola* is immunoregulatory and is depleted in rheumatoid arthritis [41], and in multiple sclerosis a *Prevotella* enterotype has been associated with lower disease severity [42]. Genus-level statements about *Prevotella* enrichment are therefore not interpretable across diseases, and species- or strain-level resolution is a precondition for valid cross-disease inference, not a refinement of it—a further argument for shotgun over amplicon sequencing where such claims are made (Section 3.1).

#### 3.1.5. Extended Marker Panels and Short-Chain Fatty Acid (SCFA) Quantification

Beyond the taxa discussed above, additional microbial markers and functional metabolite readouts strengthen multidisease classification and merit inclusion as model input features; these are collected by disease domain in Figure S1A (Supplementary Materials).

T1DM marker extensions. A pediatric systematic review of gut dysbiosis in T1DM confirmed depletion of SCFA-producing genera (*Faecalibacterium*, *Roseburia*) alongside enrichment of pro-inflammatory *Bacteroides*, *Blautia*, and *Dorea*, together with elevated gut-permeability markers zonulin and lipopolysaccharide (LPS) [43]. Bibliometric mapping of T1DM-microbiome research highlights *Faecalibacterium prausnitzii* and butyrate as an emerging citation focus [44], while tryptophan-pathway metabolites mediated by intestinal flora have been proposed as markers distinguishing rapid from slow progressors among islet-autoantibody-positive children, indicating utility as a longitudinal risk-stratification feature [45]. Mycobiome (fungal) signals are also emerging: intestinal *Candida* colonization rates are consistently elevated in T1DM patients relative to healthy controls, suggesting a candidate cross-kingdom marker for the multimodal feature set [46]. The T1DM panel is summarized in Figure S1A(a).

MOS marker extensions and SCFA mechanism. SCFAs act mechanistically through G-protein-coupled receptors FFAR2 and FFAR3, modulating the incretin hormones GLP-1 and peptide YY (PYY) and inhibiting histone deacetylases, thereby linking microbial fermentation output directly to appetite regulation and systemic inflammation [47]. A systematic review of fecal SCFA concentrations across 1650 participants found that elevated fecal SCFA levels were paradoxically associated with increased adiposity and inflammation in some cohorts, while targeted propionate or butyrate supplementation improved insulin sensitivity in interventional studies—underscoring that absolute SCFA concentration, production rate, and host receptor sensitivity must be jointly modeled rather than treated as a single linear risk marker [48]. The MOS/PCOS panel is summarized in Figure S1A(b).

CRC marker extensions. Alongside microbial taxa, clinically validated multitarget stool DNA (mt-sDNA) panels—combining methylated DNA markers (e.g., methylated ZDHHC1) with fecal hemoglobin—achieve high sensitivity and specificity for CRC and advanced precancerous lesions in large prospective cohorts, and a next-generation version of this panel (mt-sDNA 2.0, evaluated in the BLUE-C trial) reported 94% sensitivity for CRC in a study of more than 20,000 participants [49,50]. These host-genomic stool markers complement microbial taxonomic features instead of duplicating them and are a natural addition to the Host Feature MLP input in the proposed architecture; the CRC panel is summarized in Figure S1A(c).

Autoimmune disease marker extensions. A large-scale rheumatoid arthritis (RA) gut microbial biobank effort cultured 601 bacterial strains spanning 280 species directly from RA patient stool, identifying core microbial species with direct effects on host inflammation and enabling functional (not just correlative) validation of candidate marker strains [51]. *Prevotella copri* expansion has been specifically implicated in individuals at risk of developing RA prior to clinical onset, suggesting presymptomatic utility [39], while *Eubacterium rectale* has been proposed as a marker of altered gut microbiota in psoriasis and psoriatic arthritis [52]. In SLE, integrated gut-microbiota-based random forest models distinguished SLE, RA, and healthy controls with AUC = 0.792 [19], a finding restated in a recent synthesis of gut microbiota and metabolism in SLE [53], and a combined fecal cytokine and gut-barrier biomarker panel has been developed to stratify RA patients by intestinal-specific inflammatory activity [54]. The autoimmune panel is summarized in Figure S1A(d).

Figure 1 summarizes the disease-specific gut microbial marker signatures across six condition groups: T1DM; T2DM/MOS; PCOS; colorectal cancer; autoimmune disease; and dermatologic and gynecologic conditions. T1DM is shown separately from T2DM/MOS because the reported phylum-level direction differs between them (Section 3.1.1 and Section 3.1.2). The extended marker panels are provided in Table 3. and Figure S1A and the six-step fecal SCFA quantification workflow in Figure S1B (Supplementary Materials).

#### 3.1.6. Extended Disease Scope: Metabolic, Dermatologic, Gynecologic, and Additional Oncologic Associations

The multidisease framework proposed here extends naturally to further chronic and neoplastic conditions with established gut microbiome associations, broadening the diagnostic scope beyond the core T1DM/MOS/CRC/autoimmune classes.

Type 2 diabetes mellitus (T2DM). A 2024 systematic review synthesizing gut microbiota alterations across the T2DM literature confirms consistent reductions in SCFA-producing genera and expansions of opportunistic taxa paralleling the T1DM and MOS signatures described above [55]. A deep learning classifier trained on metagenomic profiles across eight disease phenotypes in more than 8000 samples achieved an AUC of 0.79 on an external T2DM cohort, confirming that the signal transfers between populations while also illustrating the magnitude of performance realistically attainable under external validation [10]. These approaches confirm that T2DM biomarker discovery follows the same metagenomic-plus-host-feature paradigm already incorporated in the proposed architecture.

Rosacea and psoriasis (gut–skin axis). Rosacea, a chronic inflammatory skin disease, is increasingly understood through the bidirectional gut–skin axis, in which gut dysbiosis, small intestinal bacterial overgrowth, and *Helicobacter pylori* infection are implicated in cutaneous inflammatory signaling [60]. Psoriasis shows a parallel and independently growing evidence base: a bibliometric mapping of the field documented an average annual growth rate of 17.9% in gut microbiota–psoriasis publications between 2021 and 2024 [61], and psoriasis shares ten gut microbiota features with other autoimmune phenotypes (RA, myasthenia gravis) captured in the multiautoimmune classifier already discussed in Section 3.1.4 [36]. Both conditions extend the autoimmune module naturally and do not require a structurally distinct classifier branch.

**Endometriosis and polycystic ovary syndrome (PCOS).** Endometriosis, a chronic gynecologic disorder involving systemic inflammation and estrogen-metabolism dysregulation, has been linked to gut dysbiosis in a large cohort study re-analyzing stool metagenomes from 1000 individuals [62], and a prospective cohort study found that combined oral, vaginal, and stool microbial signatures could serve as non-invasive diagnostic biomarkers, outperforming single-site sampling [63]. PCOS—an endocrine metabolic disorder of reproductive-aged women frequently comorbid with insulin resistance and obesity, and referred to in this project’s scope as a polyendocrine metabolic ovarian presentation—shows gut microbiota alterations acting through short-chain fatty acids, lipopolysaccharide-driven inflammation, and the gut–brain axis to influence hormone metabolism [56]. An individual-level re-analysis of 513 PCOS patients and 435 controls pooled across 14 published datasets found that gut microbiota composition distinguished PCOS from healthy controls and, notably, separated high- from low-testosterone PCOS subtypes with AUC = 0.93 [13], indicating that microbiome-based subtyping could refine the disease label beyond a single binary PCOS/healthy classification within the proposed architecture.

**Extended oncologic associations.** Beyond CRC, gut microbiome dysbiosis is implicated across the gastrointestinal cancer spectrum. Persistent dysbiosis following *Helicobacter pylori* eradication is associated with increased risk of progression to gastric intestinal-type adenocarcinoma, and stomach-resident microbiota show direct mechanistic links to gastric carcinogenesis [64]. A distinct gut microbiota signature has been shown to precede the clinical diagnosis of hepatocellular carcinoma, suggesting a presymptomatic detection window analogous to that proposed for T1DM and RA [65]. In pancreatic cancer, multiomics analyses linking gut microbiota single-nucleotide variation to tumor anatomical subtype (head vs. tail) point to microbiome-derived features with prognostic and diagnostic value even in a historically biomarker-poor malignancy [66]. A broader synthesis across colorectal, gastric, hepatobiliary, pancreatic, and esophageal cancers confirms that multiomics microbiome profiling is converging on shared methodological pipelines across GI cancer subtypes, supporting a single extensible tumor-classification branch within the disease classification module (Figure 1B) instead of a bespoke model per cancer type [67].

## 4. Proposed AI Architecture

Status of this section. No component of the architecture described below has been implemented, trained, or evaluated on data. It is a design proposal derived from the evidence synthesized in Section 3, and every performance expectation stated here is a hypothesis to be tested, not a result. The conditional is used throughout this section to mark that distinction. (Figure 2A,B)

### 4.1. Data Modalities and Preprocessing

A robust stool-based AI pipeline integrates at least three data modalities: (i) gut microbial composition from 16S rRNA amplicon sequencing or shotgun whole-genome metagenomics; (ii) predicted functional pathway abundance via PICRUSt2; and (iii) dietary pattern data from food frequency questionnaires (FFQs) or mHealth applications. Shotgun metagenomics offers strain-level taxonomic resolution and direct functional gene annotation at higher cost than amplicon sequencing; hybrid workflows combining amplicon screening with targeted shotgun sequencing in flagged samples offer a pragmatic compromise [68]. Quality control follows established amplicon pipelines (DADA2/QIIME2): demultiplexing, quality filtering, denoising, chimera removal, and taxonomic assignment against curated reference databases. Batch-effect correction (e.g., ANCOM-BC) prior to classifier training, together with nested cross-validation and leave-one-dataset-out (LODO) cross-cohort validation, is an established best practice for microbiome-based diagnostic classifiers [6]. SCFA quantification module. Functional metabolite readouts complement taxonomic and gene-level data. Gas chromatography–mass spectrometry (GC-MS) is the reference method for fecal SCFA quantification, typically after derivatization (e.g., isobutyl chloroformate or BSTFA) or, in derivatization-free protocols, direct extraction; validated methods reliably quantify acetate, propionate, butyrate, and branched-chain SCFAs (iso-butyrate, iso-valerate) with limits of detection in the low-micromolar range [69,70]. Gas chromatography with flame ionization detection (GC-FID) and liquid chromatography–tandem mass spectrometry (LC-MS/MS) with isotope-labeled internal standards offer faster, derivatization-free alternatives suitable for higher-throughput clinical pipelines [71]. Because SCFA concentrations are highly sensitive to preanalytical handling, standardized stool storage at −80 °C immediately after collection is required to prevent continued microbial fermentation and analyte degradation prior to analysis [69]. Feeding quantified SCFA profiles into the Host Feature MLP alongside taxonomic and dietary inputs allows the model to jointly learn from microbial composition and its downstream functional output. The six steps of this workflow, from stool collection to model input, are set out in Figure S1B (Supplementary Materials).

### 4.2. Proposed Multimodal Deep Learning Framework

The proposed architecture (Figure 1A,B) would integrate six complementary modules:

1. Microbiome Transformer Encoder—a self-attention transformer operating on the compositional (CLR-transformed) ASV/OTU feature matrix, capturing higher-order taxon co-occurrence patterns.
2. Dietary Embedding (BERT-food)—a language-model-based encoder fine-tuned on food composition data, converting FFQ/mHealth dietary records into a dense dietary pattern vector.
3. Host Feature MLP—a multilayer perceptron processing tabular host metadata (age, sex, BMI, HbA1c, medication status).
4. Cross-Attention Fusion Layer—Dietary Embedding outputs attend to Microbiome Transformer Encoder outputs, operationalizing the principle that diet modulates microbiome composition; the fused representation is concatenated with the host feature vector.
5. Phylo-GNN (Graph Neural Network)—a graph convolutional network operating over the phylogenetic tree of detected taxa, enabling clade-level pattern detection via message passing along evolutionary relationships.
6. Metabolic-Pathway GNN—a graph neural network operating over the reconstructed metabolic pathway network (PICRUSt2-predicted pathway abundance), yielding a pathway-level representation of community function that complements the taxonomic view.

A GAN-based data augmentation module is intended to address class imbalance across the disease categories and the healthy reference class, a persistent challenge in clinical microbiome cohorts. The fused latent vector would feed a softmax classification head producing per-class probabilities.

Incomplete modalities are the norm in this setting, not the exception: dietary logs are frequently missing or partially completed even where stool sampling succeeds. The fusion layer would therefore be trained with modality dropout, in which the dietary stream is randomly masked during training so that the model learns a usable representation from microbiome and host features alone, and a learned diet-absent embedding would substitute for the dietary vector at inference when no dietary record is available. This approach is not hypothetical in the general case: entropy-guided, sample-specific feature selection with variance-weighted fusion has been shown to maintain classification performance across missing-data patterns in gut microbiome multiomics disease prediction [72]. The magnitude of degradation under dietary absence in the present architecture is unknown and would need to be quantified explicitly, by reporting performance stratified by modality completeness, not only in complete cases.

### 4.3. Explainable AI and Microbial Risk Score

Clinical translation requires interpretability. SHAP values computed per sample identify the top contributing taxa and dietary features for each prediction; attention heatmaps from the Microbiome Transformer Encoder highlight coactivated taxon pairs. A Microbial Risk Score (MRS), a weighted combination of SHAP-selected discriminative taxa modeled on polygenic risk score methodology, provides a clinician-facing continuous risk metric that has demonstrated significant elevation in disease cohorts relative to controls in CRC screening applications [14].

The choice among explanation formats is not settled empirically. Attribution methods such as SHAP are the most widely applied in microbiome classification [5,22], and phylogeny-aware graph models have been developed specifically to make microbiome-based host phenotype prediction interpretable as well as accurate [73], which is the rationale for the Phylo-GNN component proposed here. Explainable AI has been identified as a prerequisite for clinical decision-making in microbiome-based machine learning, including in adjacent microbiome domains, alongside standardization of analytical workflows [74]. What is not established is which explanation format clinicians actually find most trustworthy for this class of model: we are not aware of a study comparing SHAP attributions, attention heatmaps and continuous risk scores for clinician trust or decision quality in a microbiome-based diagnostic setting. Risk scores have the pragmatic advantage of matching a format already in clinical use, but this is an argument from familiarity rather than from evidence, and the question warrants dedicated human-factors study.

## 5. Discussion

### 5.1. Dietary Integration and Future Directions

Diet is the primary short-timescale determinant of gut microbiome composition, making it an essential covariate in microbiome-based disease classifiers [7]. Deep learning models incorporating dietary inputs alongside microbiome profiles have been shown to improve prediction of individual glycemic and metabolic responses beyond what microbiome data alone can achieve [75]. Precision nutrition seeks to tailor dietary guidance to individual biological and behavioral characteristics instead of population averages, and the gut microbiome has become one of the principal axes along which such tailoring is attempted [7]. AI-enabled personalized nutrition delivered through mobile and digital tools has been proposed as a route to modulate the microbiome, with short-term improvements in diversity and anthropometric measures summarized in recent reviews [76]. An AI-powered digital gut twin concept has been proposed to simulate dietary interventions and their downstream effects on microbiome composition and cancer risk trajectories, illustrating a plausible translational pathway for the broader multidisease framework proposed here [7].

The magnitude of the contribution that dietary data make to microbiome-based classification is smaller than is often assumed and is rarely quantified directly. Where it has been, the increment was statistically significant but numerically modest: adding a dietary index to a baseline model changed the AUC from 0.741 to 0.743 (*p* = 0.013) [24]. To our knowledge no study has reported a with-diet versus without-diet difference in AUC for disease classification on the same cohort, and we therefore state the expected benefit of the dietary module as a testable prediction, not an established result. A plausible explanation for the small observed increment is that much of the dietary signal is already encoded in the fecal sample itself: fecal metabolite profiles predict reported intake across twenty food groups with AUC above 0.80 [77]. Dietary questionnaires may therefore add less information beyond the stool sample than the conceptual separation of the two modalities suggests. This is an argument for measuring the incremental contribution of each modality explicitly, by ablation within a single pipeline, instead of assuming it.

These estimates are further constrained by the difficulty of measuring dietary intake at all. Self-reported instruments—food frequency questionnaires, 24 h recalls, diet records—are subject to substantial recall bias and measurement error, which limits their precision and clinical utility [78]; methodological and reproducibility challenges specific to diet–microbiome research have been characterized in detail [79]. Instruments developed specifically for microbiome studies exist [80], and technology-assisted approaches including image-based logging, wearable sensors and AI-based intake estimation may reduce reliance on recall [78], but between-instrument variability remains a source of heterogeneity that any multimodal model inherits from its inputs.

Priority research directions follow from these constraints: longitudinal validation in presymptomatic cohorts, standardized biobanking and preanalytical protocols, and federated learning approaches that enable multi-institutional model training without centralizing patient-level data—the last of these addressing directly the data-protection barrier set out in Section 5.2.

### 5.2. Limitations of the Evidence Base and Barriers to Clinical Translation

Risk of bias in the evidence base. A PROBAST-informed appraisal of the studies in Table 1 (Table S2) found no study at low overall risk of bias and identified two limitations that recur across the field instead of attaching to individual studies. First, only one of the fifteen appraised studies reported any measure of calibration [14]; the remainder characterized performance by discrimination alone. This matters because a classifier that discriminates well but is poorly calibrated assigns miscalibrated absolute risks, and absolute risk, not rank ordering, is what a screening application must deliver. A targeted search of the same disease domains did not identify further microbiome-based detection or screening models reporting calibration, although abstract-level searching will under-detect calibration reported only in a Section 3. Second, every appraised study evaluated classifiers in populations in which the disease was already diagnosed or clinically apparent, typically under a case–control design; applicability to the presymptomatic screening setting proposed here was judged to be of high concern in thirteen of the fifteen studies. A recent meta-analysis of fecal SCFA in colorectal cancer reaches the same conclusion from a different direction, noting that ten of its thirteen included studies performed no prediagnostic exposure assessment [57].

It is notable that the single study reporting calibration was also the one explicitly designed as a screening tool rather than as a classifier benchmark [14]. Studies framed as benchmarks, typically built by re-analyzing pooled public case–control cohorts, report discrimination because discrimination is what a benchmark compares; studies framed as clinical prediction instruments report calibration and clinical utility because those are what an instrument must demonstrate. The literature underpinning the framework proposed here belongs overwhelmingly to the first category. Adopting the reporting conventions of the second—calibration, decision curve analysis, and prespecified external validation, in line with TRIPOD recommendations for prediction model reporting and with emerging minimum reporting frameworks for machine-learning-based nutritional prediction [81]—is a low-cost and immediately actionable change and a precondition for evaluating any of these models as a screening instrument rather than as a demonstration of biological signal.

Analytical standardization and inter-laboratory reproducibility. As shown in Section 3.1, pipeline choice within a single sequencing technology can account for a wider performance spread than the difference between technologies [15]. Deployment across laboratories therefore requires prespecified, version-controlled pipelines covering DNA extraction, sequencing chemistry, taxonomic reference database, batch correction and normalization, not merely agreement on the sequencing platform. Confounding by diet, host genetics, geographic origin and medication use shapes microbiome signatures independently of disease and must be handled at the design stage, not by post hoc adjustment [79].

Regulatory and economic barriers. Metagenomic data are sensitive health information, and a screening deployment would require an explicit regulatory pathway for microbiome-based diagnostic software, demographic representativeness of training cohorts, and a cost per screen compatible with population-scale application. None of these has been established for the multidisease setting.

What would change the assessment. The evidence reviewed here establishes that fecal microbial signatures carry disease-discriminating information; it does not establish that they carry that information before clinical presentation, in a screening population, at a calibrated absolute risk. Closing that gap requires prospective enrolment of asymptomatic individuals with longitudinal follow-up to incident diagnosis, prespecified analysis pipelines, and reporting of calibration and net benefit alongside discrimination. It is not addressable by improved model architecture alone.

## 6. Conclusions

The convergence of stool metagenomics, dietary data capture and multimodal deep learning defines a plausible research pathway toward population-scale screening for T1DM, MOS, CRC and autoimmune disease in their presymptomatic phase. The evidence reviewed indicates that microbiome-based classifiers achieve AUCs spanning 0.76–0.99 under internal validation, falling to 0.69–0.91 under external or cross-population validation, with the median of the four paired estimates falling from 0.875 to 0.810; that disease-specific signatures are reproducible across independent cohorts for intestinal disease but transfer considerably less well elsewhere; that integrating metabolite measurements improves discrimination measurably whereas the directly quantified contribution of dietary data has so far been small; and that explainability tools are a necessary but not sufficient precondition for clinical adoption. The multimodal framework proposed here—combining a microbiome transformer, a dietary language-model encoder, a phylogenetic graph neural network and cross-attention fusion—is offered as an architecturally grounded design hypothesis, not as a validated system. Continuous re-training on accumulating clinical outcome data is built into the design as an improvement mechanism, but its behavior is untested: adaptation would need to be evaluated prospectively against calibration drift, with a prespecified re-training cycle, monitoring of performance by subgroup, and a rollback criterion, none of which can be inferred from the retrospective, cross-sectional studies that constitute the present evidence base. For slowly evolving autoimmune phenotypes in particular, the interval over which such a mechanism could demonstrate benefit is measured in years, and no cohort of the required design currently exists. The field is, in short, technically feasible and clinically unproven. The decisive next step is not a better architecture but a prospective, harmonized, calibration-reporting trial in an asymptomatic population.

## Figures and Tables

**Table 1:** Reported diagnostic performance of gut-microbiome-based machine learning classifiers across the reviewed disease domains. Where a study reported both internal and external validation, both values are given. NR = not reported; LODO = leave-one-dataset-out; CV = cross-validation. The four studies contributing an internal/external pair to the median reported in Section 3.1 are Tsai et al. (0.90 to 0.82), Sun et al. (0.85 to 0.86, optimized pipeline under LODO), Li M et al. (0.77 to 0.73) and Su et al. (0.945 to 0.800, midpoints of the reported ranges); midpoints are used wherever a study reports a range instead of a single value.

Study/Disease Domain/Cohort	Sequencing	AI Model	Validation	AUC	Population(s)	Principal Limitation
Chavarria et al. 2025 [8] T1DM/T2DM screening n = NR	16S (hypervariable features)	Random forest, SVM	Internal CV	0.76 (0.77 with 500 features)	Public repository	Authors state all models lacked sensitivity; diabetes status self-reported
Tan et al. 2024 [9] T1DM (multiomics) n = 137 (38 + 38 + 61)	16S + metabolomics + lipidomics	Multiomics ML	Internal	NR	Chinese	Very low events-per-variable; no external validation
Li J et al. 2025 [10] T2DM n = >8000; 8 phenotypes	Metagenomics	Deep deformable CNN	Within-cohort + external	0.7921 (external T2D cohort)	Multicohort	Deep model; interpretability limited
Wu et al. 2024 [11] Obesity/MOS n = 1563	Shotgun	XGBoost (top-40 species)	Internal CV	NR (accuracy only)	Chinese	AUC not reported; single population
Zhao et al. 2026 [12] Overweight (multiomic) n = NR	Metagenomics + metabolomics	Random forest	Internal	Microbiota >0.70 to 0.818 with metabolites	Mongolian	Single population; the with/without contrast is the informative result
Yang et al. 2024 [13] PCOS (incl. subtyping) n = 513/435; 14 datasets	16S/shotgun (pooled)	Individual-level pooled analysis	Cross-dataset	0.93 (high- vs. low-testosterone subtype)	Pooled international	Re-analysis of published data; PCOS criteria differ across datasets
Tsai et al. 2025 [14] CRC + adenoma n = 5 datasets	16S	Random forest	Internal CV + external	0.90 (95% CI 0.869–0.931) to 0.82	North American + East Asian	Only study reporting calibration (Brier score, calibration slope)
Sun et al. 2025 [15] CRC + adenoma n = 4217	16S vs. shotgun, head-to-head	6468 pipelines; XGBoost/RF best	5-fold CV + LODO	CRC: WGS 0.85 vs. 16S 0.82; averaged 0.86 vs. 0.77. Adenoma: 0.73 vs. 0.67	AT, CN, DE, IT, JP, IN, FR, ES, US	Methodological benchmark, not a prospective trial
Piccinno et al. 2025 [16] CRC n = 3741 metagenomes; 18 cohorts	Shotgun	Pooled ML meta-analysis	Cross-cohort, strain-level	Cross-stage reproducible biomarkers	18 international cohorts	Retrospective pooling of published cohorts
Gao et al. 2023 [17] CRC (SNV markers) n = 3 independent validation cohorts	Shotgun (SNV)	ML	Three external cohorts	0.83/0.82/0.76	Multicohort	Sensitivity 1.00/0.72/0.93, specificity 0.67/0.81/0.67: unstable operating characteristics
Okumura et al. 2025 [18] CRC screening vs. FIT n = 81 CRC/245 healthy	16S	ML gut microbiota model, fixed cut-off	Reproducibility test vs. FIT	TPR 53.1% vs. FIT 86.4%; FPR 7.3% vs. FIT 2.4%	Japanese	Combining with FIT raises detection to 91.4% but FPR to 9.4%
Zheng et al. 2024 [4] IBD (UC, CD) n = 5979	Shotgun	10-species (UC)/9-species (CD)	Discovery + transethnic (8 populations)	>0.90 in discovery; maintained transethnically	Multiple geographies and ethnicities	Cross-sectional; no presymptomatic cohort
Li Y et al. 2019 [19] SLE vs. RA vs. controls n = NR	16S	Random forest	Internal	0.792 (SLE activity 0.811)	Chinese	Predates the survey window; cited as primary source (Section 2.2)
Li M et al. 2023 [20] 20 diseases (cross-cohort) n = 69 projects	16S/shotgun	ML classifiers, nested CV	Intra- vs. cross-cohort	~0.77 intra to ~0.73 cross (intestinal only)	Multiple	Non-intestinal disease signals do not transfer
Su et al. 2022 [21] Multiclass disease n = 2320 + 1597 external	Fecal 16S/metagenomics	ML, nested CV	Independent test + external public	0.90–0.99 to 0.69–0.91	Hong Kong + international	Largest internal-to-external gap in the table

**Table 2:** Gut microbial taxa reported as enriched or depleted across the chronic conditions reviewed, with an assessment of whether the reported direction is consistent across independent studies. Conditions are included whether or not they appear in Figure 1. The source for each entry is the study cited for that taxon in the corresponding subsection of Section 3.1; entries are not re-cited here to keep the table readable.

Condition	Enriched	Depleted	Direction Consistent Across Studies?
T1DM	Bacteroides, Blautia, Dorea; Bacteroidetes (phylum)	Firmicutes; *Faecalibacterium prausnitzii*, *Roseburia*, *Akkermansia muciniphila*	Yes in children; age-dependent (see Section 3.1.1)
T1DM (mycobiome)	Intestinal *Candida* colonization	-	Limited; few studies, small cohorts
T2DM	Opportunistic taxa	SCFA-producing genera	Partly; genus-level replicates, phylum-level ratio does not
MOS/obesity	Firmicutes (contested)	Bacteroidetes (contested); *A. muciniphila*, *Bifidobacterium pseudocatenulatum*, *Lactobacillus*	No at phylum level; genus-level findings more robust
PCOS	LPS-associated taxa	SCFA producers	Partly; separates PCOS from controls and stratifies testosterone subtypes
Colorectal cancer	*Fusobacterium nucleatum*, *Parvimonas micra*, *Peptostreptococcus anaerobius*, *Bacteroides fragilis*	Butyrate producers	Yes; the most reproducible signature in the field
Gastric cancer	Dysbiosis persisting after *H. pylori* eradication	-	Emerging
Hepatocellular carcinoma	Signature preceding clinical diagnosis	-	Single-cohort; presymptomatic window suggested, not validated
Pancreatic cancer	Microbiota single-nucleotide variation by anatomical subtype	-	Exploratory
Breast cancer	Deoxycholic-acid-related taxa	-	Single study
Rheumatoid arthritis	*Prevotella copri* (species level, incl. preclinical); *Haemophilus* spp.	Butyrate producers; Prevotella histicola	Yes for *P. copri* in RA; the preclinical evidence is antibody-based
SLE	*Prevotella* spp. (genus level), *Eggerthella*	Butyrate producers	Genus-level only; see note in Section 3.1.4
IBD—Crohn’s disease	*Haemophilus* spp.	-	Yes; 9-species model, transethnic validation
IBD—ulcerative colitis	*Haemophilus* spp.	-	Yes; 10-species model, transethnic validation
Psoriasis/psoriatic arthritis	*Prevotella copri*	*Eubacterium rectale*, butyrate producers	Partly; shares ten features with RA and myasthenia gravis
Multiple sclerosis	*Prevotella enterotype* (associated with LOWER severity)	-	Direction opposite to RA; see Section 3.1.4
Myasthenia gravis	-	Butyrate producers	Limited; identified within a multiautoimmune classifier
Rosacea	SIBO, Helicobacter pylori association	-	Mechanistic hypothesis; no classifier reported
Endometriosis	Multisite (oral, vaginal, stool) signature	-	Partly; combined-site sampling outperforms single-site

**Table 3:** Short-chain fatty acid and related metabolite alterations reported across the reviewed conditions, with measurement method and interpretive caveat.

Condition	Metabolite/Functional Readout	Reported Direction	Measurement	Caveat
T1DM	Butyrate; total SCFA	Reduced (via depletion of Faecalibacterium, *Roseburia*)	Inferred from taxonomy; GC-MS where measured [43]	Mostly inferred from producer abundance, not directly quantified
T1DM	Zonulin, lipopolysaccharide	Increased	ELISA [43]	Gut-permeability markers, not microbial products per se
T1DM (progression)	Tryptophan-pathway metabolites	Differentiates rapid vs. slow progressors	LC-MS [45]	Longitudinal risk stratification, not diagnosis
T2DM	SCFA-producing capacity	Reduced (consistent depletion of SCFA-producing genera)	Systematic review synthesis [55]	Inferred from producer abundance; not directly quantified
MOS/obesity	Fecal SCFA (acetate, propionate, butyrate)	Higher fecal concentration associated with greater adiposity in some cohorts	GC-MS, 1650 participants [48]	Paradoxical: fecal concentration reflects production minus absorption, not production rate
MOS/obesity	Propionate, butyrate (supplementation)	Improved insulin sensitivity	Interventional [48]	Opposite direction to the observational finding above
MOS mechanism	FFAR2/FFAR3 signaling; GLP-1, PYY; HDAC inhibition	-	[47]	Mechanistic pathway linking fermentation output to appetite and inflammation
PCOS	SCFA; LPS	SCFA reduced; LPS increased	Mixed [56]	Acts via gut–brain axis on hormone metabolism
Colorectal cancer	Fecal acetate	Reduced (pooled SMD −0.37; 95% CI −0.63 to −0.10; I^2^ = 0%)	GC-MS, meta-analysis [57]	Four studies pooled; 141 CRC cases
Colorectal cancer	Fecal butyrate	Reduced (pooled SMD −0.59; 95% CI −1.10 to −0.07; I^2^ = 64.4%)	GC-MS, meta-analysis [57]	Moderate heterogeneity
Colorectal cancer	Fecal propionate	No significant difference (SMD −0.02; I^2^ = 89%)	GC-MS, meta-analysis [57]	Very high heterogeneity; pooled estimate not interpretable
Colorectal cancer	Total SCFA vs. CRC risk	OR 0.78 (95% CrI 0.65–0.92); advanced adenoma OR 0.72	Bayesian meta-analysis, 14 studies [58]	Fecal OR 0.73 vs. serum/plasma OR 0.85: sample type matters
Colorectal cancer	Fecal acetate as single marker	AUC 0.843 for CRC diagnosis	1H NMR metabolomics [59]	Training set; validation retained predictive ability
Colorectal cancer	Methylated ZDHHC1 + fecal hemoglobin (mt-sDNA)	Increased	Multitarget stool DNA assay [49,50]	Host-genomic, complementary to microbial features; 94% sensitivity in >20,000 participants
Colorectal cancer	Combined 5 microbial + 5 metabolite panel	-	LC-MS + 16S [23]	AUC 0.85 for adenoma vs. carcinoma, exceeding CEA and CA19-9 specificity
Overweight	Multikingdom microbiota + metabolites	-	Metagenomics + metabolomics [12]	Microbiota alone AUC >0.70 to 0.818 with metabolites: a direct with/without contrast
Breast cancer	Deoxycholic acid	Increased	[35]	Bile-acid rather than SCFA pathway
Rheumatoid arthritis	Fecal cytokines + gut-barrier biomarkers	Increased	Panel assay [54]	Stratifies intestinal-specific inflammatory activity

## Data Availability

No new data were created or analyzed in this study. Data sharing is not applicable to this article.

## Supplementary Materials

The following supporting information can be downloaded at https://www.mdpi.com/article/10.3390/microorganisms14091880/s1. Table S1. Complete literature search record. Table S2. PROBAST-informed risk-of-bias and applicability appraisal. Risk of bias and applicability were appraised independently by two authors using a PROBAST-informed framework across the four PROBAST domains (participants, predictors, outcome, analysis), based on the full texts of all 15 studies in Table 1. The two assessments agreed on every appraised study. L = low, M = moderate, H = high, U = unclear, NA = not assessed. Figure S1. Extended disease-specific marker panels and fecal short-chain fatty acid (SCFA) quantification workflow for multi-disease microbiome classification. (A) Extended biomarker panels by disease domain: (a) T1DM—depleted SCFA producers, enriched *Bacteroides*/*Blautia*/*Dorea*, gut-permeability markers and mycobiome signal, reported at genus level only, the phylum-level summary being given in Figure 2; (b) MOS and PCOS—depleted *Akkermansia muciniphila*, *Bifidobacterium pseudocatenulatum* and *Lactobacillus*, fecal SCFA, FFAR2/FFAR3 engagement and GLP-1/PYY incretin coupling, with the PCOS-specific pattern of reduced SCFA, elevated LPS and altered testosterone signalling; (c) colorectal cancer—*Fusobacterium nucleatum*, the methylated ZDHHC1 multi-target stool DNA panel, fecal hemoglobin and a 109-taxon random-forest signature, with the fecal immunochemical test (FIT) as comparator and its false-positive rate rising from 2.4% to 9.4% when combined with microbiome features; (d) autoimmune disease—*Prevotella copri* at species level in rheumatoid arthritis and pre-symptomatic rheumatoid arthritis, distinguished from genus-level *Prevotella* spp. in systemic lupus erythematosus, together with depleted *Eubacterium rectale*, the core rheumatoid-arthritis biobank species, a fecal cytokine and gut-barrier panel and the 10/9-species inflammatory bowel disease diagnostic panels. (B) Six-step fecal SCFA quantification pipeline: stool collection with standardized storage at −80 °C immediately after collection, direct extraction from stool, optional derivatization (isobutyl chloroformate or BSTFA, omitted in derivatization-free protocols), instrumental analysis by GC-MS as the reference method or by GC-FID or LC-MS/MS with isotope-labeled internal standards, the quantified profile of acetate, propionate, butyrate and the branched-chain SCFAs iso-butyrate and iso-valerate, and integration as Host Feature MLP input alongside taxonomic and dietary embeddings. Cited in Section 3.1.5 and Section 4.1; see Table 2 for the full disease list.

## Abbreviations

The following abbreviations are used in this manuscript: AI, artificial intelligence; ASV, amplicon sequence variant; AUC, area under the receiver operating characteristic curve; CLR, centered log-ratio; CRC, colorectal cancer; CV, cross-validation; FFQ, food frequency questionnaire; FIT, fecal immunochemical test; GAN, generative adversarial network; GC-FID, gas chromatography with flame ionization detection; GC-MS, gas chromatography–mass spectrometry; GNN, graph neural network; HCC, hepatocellular carcinoma; IBD, inflammatory bowel disease; LC-MS/MS, liquid chromatography–tandem mass spectrometry; LODO, leave-one-dataset-out; LPS, lipopolysaccharide; ML, machine learning; MLP, multilayer perceptron; MOS, metabolic obesity syndrome; MRS, Microbial Risk Score; mt-sDNA, multitarget stool DNA; OTU, operational taxonomic unit; PCOS, polycystic ovary syndrome; PROBAST, Prediction model Risk Of Bias ASsessment Tool; RA, rheumatoid arthritis; SCFA, short-chain fatty acid; SHAP, Shapley additive explanations; SLE, systemic lupus erythematosus; SMD, standardized mean difference; T1DM, type 1 diabetes mellitus; T2DM, type 2 diabetes mellitus; TRIPOD, Transparent Reporting of a multivariable prediction model for Individual Prognosis Or Diagnosis.
