# Supplementary material for: Toward AI-Driven Detection of Asymptomatic Chronic Conditions from Stool Metagenomics and Dietary Data: A Multimodal Deep Learning Framework for T1DM, T2DM, MOS/PCOS, Cancer, and Autoimmune Disease
Source: Microorganisms. 2026 Aug 24;14(9):1880. doi: 10.3390/microorganisms14091880 (PMC13609121; doi:10.3390/microorganisms14091880)
Supplement: Supplementary file 1 [file microorganisms-14-01880-s001.zip › microorganisms-4452264-supplementary.pdf]

Supplementary Materials

Toward AI-Driven Detection of Asymptomatic Chronic Conditions from Stool Metagenomics and Dietary Data: A Multimodal Deep Learning Framework for T1DM, T2DM, MOS/PCOS, Cancer, and Autoimmune Disease

Szili K., Dézsi C., Gulyás-Oldal V., Sallai D., Patay G., Paschali E., Nagy S. — Microorganisms, manuscript ID microorganisms-4452264

Table S1. Complete literature search record

Topic (evidence base) — 31 searches, 252 result items

#	Source	Query	Hits
1	web	AI machine learning gut microbiome detection asymptomatic chronic disease T1DM cancer autoimmune 2022 2023 2024	7
2	web	fecal microbiome PCOS type 1 diabetes autoimmune early detection machine learning stool 2023 2024	7
3	web	gut microbiome colorectal cancer early detection AI deep learning fecal stool 2023 2024 2025	8
4	web	dietary data integration microbiome machine learning disease prediction precision nutrition 2023 2024	8
5	web	gut microbiome PCOS polycystic ovary syndrome dysbiosis biomarker machine learning 2023 2024 2025	8
6	web	multiclass disease classification stool metagenomics deep learning transformer multi-disease 2023 2024 2025	9
7	web	machine learning gut microbiome multiclass disease detection stool metagenomics 2023 2024 2025	9
8	web	T1DM gut microbiome dysbiosis machine learning autoimmune detection 2022 2023 2024	7
9	web	metabolic obesity syndrome gut microbiome Akkermansia insulin resistance AI classification 2022 2023 2024	9
10	web	gut microbiome colorectal cancer AI stool metagenomics early detection AUC 2023 2024 2025	7
11	web	precision nutrition diet microbiome deep learning AI disease prevention 2023 2024 2025	8

12	web	gut microbiome host metabolism immunity environment interface review 2022 2023	8
13	web	16S rRNA amplicon versus shotgun metagenomics sequencing comparison cost taxonomic resolution review 2022 2023	8
14	web	Greathouse Precision nutrition gut microbiome AI cancer prevention digital twin Cell Host Microbe 2025	7
15	web	\"human gut microbiota is associated with host lifestyle\" comprehensive narrative review Frontiers Microbiology authors	5
16	web	T1DM stool microbiome biomarker panel specific bacterial strains 2023 2024 2025	9
17	web	short-chain fatty acid SCFA measurement method GC-MS stool gut microbiome 2023 2024	9
18	web	colorectal cancer multi-marker stool panel biomarker combination AI 2024 2025	9
19	web	metabolic syndrome obesity gut microbiome marker strain SCFA butyrate propionate 2023 2024	7
20	web	rheumatoid arthritis lupus gut microbiome marker strain biomarker panel 2023 2024	9
21	web	Xiao Eubacterium rectale marker altered gut microbiota psoriasis psoriatic arthritis journal PMID 38239365	9
22	web	Prevotella copri individuals at risk rheumatoid arthritis 2023 2024 recent study	9
23	web	Lamichhane Sen Dickens circulating metabolic signatures rapid slow progression type 1 diabetes islet autoantibody Frontiers Endocrinology	8
24	web	\"Gas chromatography-mass spectrometry\" fecal short-chain fatty acids summary review own experience Nutrients volume	5
25	web	type 2 diabetes gut microbiome AI classification biomarker 2023 2024 2025	10
26	web	rosacea gut microbiome dysbiosis skin-gut axis 2023 2024 2025	9
27	web	psoriasis gut microbiome biomarker machine learning 2023 2024 2025	8
28	web	endometriosis gut microbiome biomarker diagnosis 2023 2024 2025	9
29	web	PCOS polycystic ovary	8

		syndrome gut microbiome machine learning biomarker 2023 2024 2025	
30	web	gastric cancer pancreatic cancer gut microbiome stool biomarker AI 2023 2024 2025	10
31	web	Lamichhane circulating metabolic signatures islet autoantibody-positive children type 1 diabetes Frontiers Endocrinology 2023	9

Verification (revision) — 23 searches, 4622 result items

#	Source	Query	Hits
1	pubmed	type 1 diabetes Firmicutes Bacteroidetes ratio gut microbiota	29
2	pubmed	Firmicutes Bacteroidetes ratio obesity meta-analysis reappraisal hallmark	669
3	pubmed	(16S[tiab] AND (shotgun[tiab] OR metagenomic*[tiab])) AND ("machine learning"[tiab] OR classifier*[tiab] OR "random forest"[tiab]) AND (AUC[tiab] OR AUROC[tiab] OR "area under"[tiab])	58
4	pubmed	(diet*[tiab] OR nutrition*[tiab]) AND microbiom*[tiab] AND ("machine learning"[tiab] OR classifier*[tiab]) AND (AUC[tiab] OR AUROC[tiab]) AND (improv*[tiab] OR integrat*[tiab])	19
5	pubmed	((SCFA*[tiab] OR "short-chain fatty acid*"[tiab] OR metabolom*[tiab]) AND microbiom*[tiab]) AND ("colorectal cancer"[tiab] OR CRC[tiab]) AND (AUC[tiab] OR specificity[tiab] OR "false positive"[tiab])	22
6	pubmed	(clinician*[tiab] OR physician*[tiab]) AND (trust[tiab] OR preference*[tiab]) AND ("explainable AI"[tiab] OR XAI[tiab] OR interpretab*[tiab] OR SHAP[tiab])	537
7	pubmed	(microbiom*[tiab] OR microbiota[tiab]) AND diet*[tiab] AND ("addition of"[tiab] OR "improved from"[tiab]) AND (AUC[tiab] OR AUROC[tiab])	6
8	pubmed	(microbiom*[tiab] OR metagenom*[tiab]) AND ("multi-disease"[tiab] OR "across diseases"[tiab]) AND (AUC[tiab] OR classifier*[tiab]) AND 2021:2026[dp]	5
9	pubmed	("colorectal cancer"[tiab]) AND (metagenom*[tiab] OR microbiom*[tiab]) AND ("meta-analysis"[tiab] OR "cross-cohort"[tiab]) AND (AUC[tiab] OR AUROC[tiab])	13
10	pubmed	Zheng noninvasive microbiome-based diagnosis inflammatory bowel disease Nature Medicine 2024	3
11	pubmed	Piccinno G[au] AND colorectal	10
12	pubmed	(microbiom*[tiab] OR microbiota[tiab] OR metagenom*[tiab]) AND ("Brier"[tiab] OR "calibration curve"[tiab] OR "calibration slope"[tiab] OR "Hosmer-Lemeshow"[tiab] OR "well-calibrated"[tiab] OR "decision curve"[tiab])	55
13	pubmed	(microbiom* OR microbiota OR metagenom*) AND (CRC|T1DM/T2DM|IBD/autoimmun|obezitas/MOS/PCOS|GI-daganat) AND (Brier|calibration curve|slope|Hosmer-Lemeshow|nomogram) AND (diagnos*|screen*|detect*)	21
14	pubmed	(microbiom*[tiab] OR microbiota[tiab] OR metagenom*[tiab]) AND (SHAP[tiab] OR "explainable"[tiab] OR interpretab*[tiab]) AND (diagnos*[tiab] OR classif*[tiab] OR screen*[tiab])	270
15	pubmed	(Prevotella[tiab]) AND (lupus[tiab] OR SLE[tiab] OR "rheumatoid arthritis"[tiab] OR psorias*[tiab] OR "multiple sclerosis"[tiab] OR autoimmun*[tiab]) AND (gut[tiab] OR fecal[tiab] OR microbiot*[tiab])	286
16	pubmed	(microbiom*[tiab] OR microbiota[tiab]) AND ("food frequency questionnaire"[tiab] OR "dietary assessment"[tiab] OR "24-hour recall"[tiab]) AND (limitation*[tiab] OR bias[tiab] OR validity[tiab] OR variability[tiab])	31

17	pubmed	(microbiom*[tiab] OR microbiota[tiab] OR metagenom*[tiab]) AND ("inter-laboratory"[tiab] OR standardi*[tiab] OR reproducib*[tiab] OR regulatory[tiab] OR "clinical translation"[tiab]) AND (diagnos*[tiab] OR biomarker*[tiab])	2332
18	pubmed	("fecal immunochemical test"[tiab] OR FIT[tiab]) AND (microbiom*[tiab] OR metabolit*[tiab] OR SCFA*[tiab]) AND ("false positive"[tiab] OR specificity[tiab] OR unnecessary[tiab])	88
19	pubmed	("unnecessary colonoscop*"[tiab] OR triage[tiab] OR "rule-out"[tiab]) AND (stool[tiab] OR fecal[tiab]) AND (microbiom*[tiab] OR metabolom*[tiab])	29
20	pubmed	(SCFA*[tiab] OR metabolom*[tiab]) AND microbiom*[tiab] AND colorectal[tiab] AND ("addition of"[tiab] OR "combined model"[tiab] OR "integrated model"[tiab]) AND (specificity[tiab] OR AUC[tiab])	1
21	pubmed	("short-chain fatty acid*"[tiab] OR SCFA*[tiab] OR butyrate[tiab]) AND (fecal[tiab] OR stool[tiab]) AND ("colorectal cancer"[tiab] OR adenoma[tiab]) AND (sensitivity[tiab] OR specificity[tiab] OR AUC[tiab])	7
22	pubmed	("type 1 diabetes"[tiab]) AND (Firmicutes[tiab] AND Bacteroidetes[tiab]) AND (gut[tiab] OR fecal[tiab] OR intestinal[tiab])	45
23	pubmed	("Firmicutes/Bacteroidetes"[tiab] OR "F/B ratio"[tiab]) AND (limitation*[tiab] OR criticis*[tiab] OR unreliab*[tiab] OR question*[tiab] OR confound*[tiab])	86

Journal selection (not part of the evidence base) — 13 searches, 119 result items

#	Source	Query	Hits
1	web	gut microbiome AI review journal Q2 impact factor 2024 open access	8
2	web	Norwegian Register Scientific Journals level list meaning exclude predatory	7
3	web	\"Frontiers in Cellular and Infection Microbiology\" impact factor quartile 2024	10
4	web	Microbiology Spectrum ASM journal impact factor quartile 2024 review article	10
5	web	Gut Pathogens journal impact factor quartile SJR ranking 2024	7
6	web	MDPI journal gut microbiome AI disease review impact factor 2024 comparison	9
7	web	International Journal of Molecular Sciences Nutrients Biomedicines impact factor 2024 quartile	10
8	web	Nutrients journal MDPI impact factor 2024 quartile Microorganisms impact factor 2024	9
9	web	Microorganisms MDPI acceptance rate article processing charge APC 2025	10
10	web	Nutrients MDPI acceptance rate APC publication fee 2025	10
11	web	Biomedicines MDPI acceptance rate APC publication fee 2025	10
12	web	Gut Pathogens BMC article processing charge acceptance rate 2025	9

13	web	Frontiers Cellular Infection Microbiology acceptance rate article processing charge 2025	10

Table S2. PROBAST-informed risk-of-bias and applicability appraisal

Risk of bias and applicability were appraised independently by two authors using a PROBAST-informed framework across the four PROBAST domains (participants, predictors, outcome, analysis), based on the full texts of all 15 studies in Table 1. The two assessments agreed on every appraised study. L = low, M = moderate, H = high, U = unclear, NA = not assessed.

**Study**	**Participants**	**Predictors**	**Outcome**	**Analysis**	**Overall RoB**	**Applicability**
Chavarria 2025	H	U	H	H	H	H
Tan 2024	H	L	L	H	H	H
Wu 2024	U	L	L	H	H	H
Zhao 2026	U	L	L	H	H	H
Tsai 2025	H	U	L	L	M	H
Sun 2025	H	L	L	L	M	H
Piccinno 2025	H	L	L	M	M	H
Gao 2023	H	U	L	H	H	H
Zheng 2024	H	L	L	M	M	H
Li Y 2019	H	L	H	L	H	H
Yang 2024	H	U	U	M	H	H
Li M 2023	H	U	U	L	M	M
Su 2022	M	L	L	L	M	H
Li J 2025	H	U	U	M	H	H
Okumura 2025	M	L	L	M	M	M

Summary: no study was at low overall risk of bias (0/15); one study reported any measure of calibration (1/15); applicability to pre-symptomatic screening was of high concern in 13 of the 15 studies.

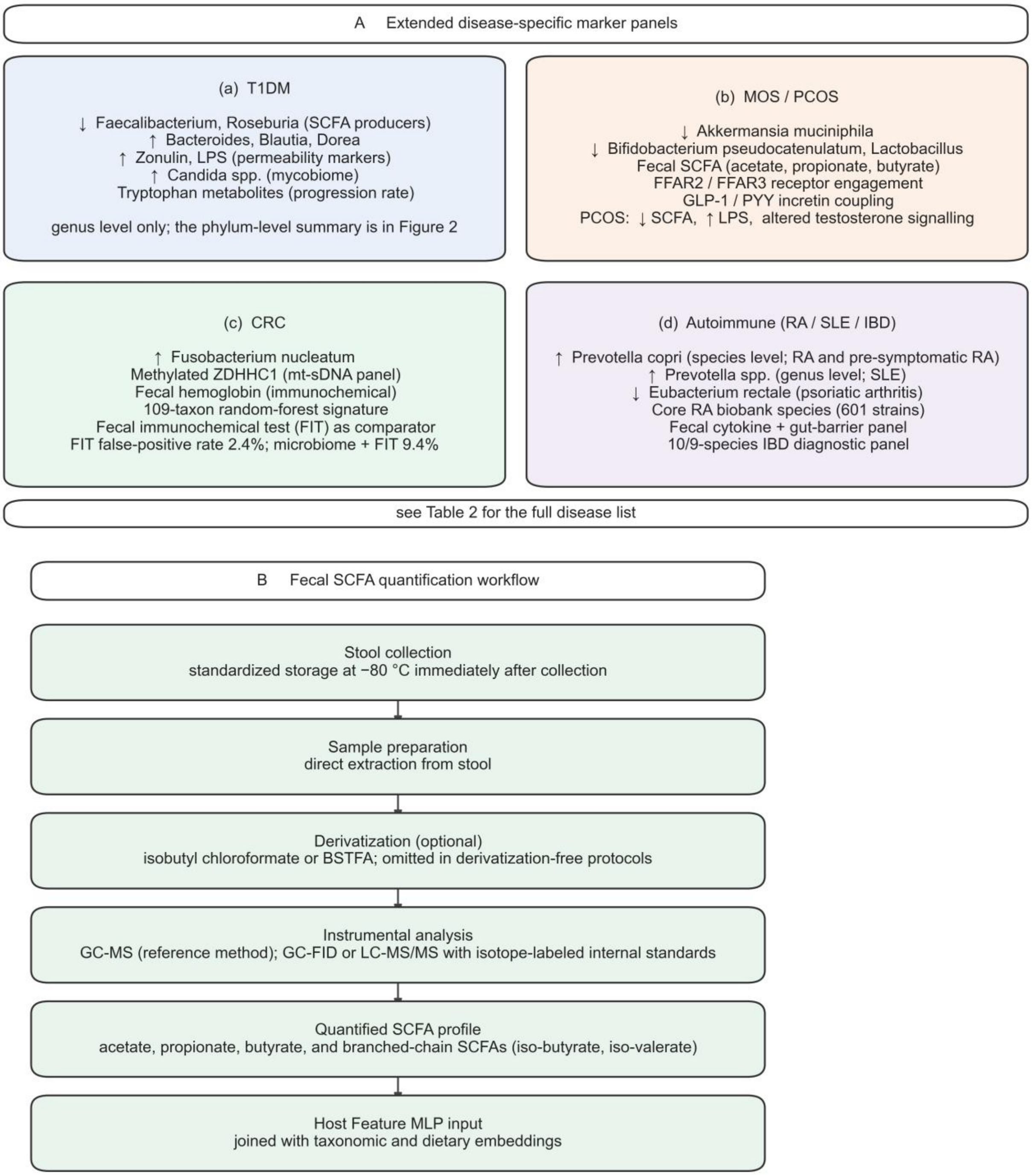

Figure S1. Extended disease-specific marker panels and fecal short-chain fatty acid (SCFA) quantification workflow for multi-disease microbiome classification. (A) Extended biomarker panels by disease domain: (a) T1DM - depleted SCFA producers, enriched Bacteroides/Blautia/Dorea, gut-permeability markers and mycobiome signal, reported at genus level only, the phylum-level summary being given in Figure 2; (b) MOS and PCOS - depleted Akkermansia muciniphila, Bifidobacterium pseudocatenulatum and Lactobacillus, fecal SCFA, FFAR2/FFAR3 engagement and GLP-1/PYY incretin coupling, with the PCOS-specific pattern of reduced SCFA, elevated LPS and altered testosterone signalling; (c) colorectal cancer - Fusobacterium nucleatum, the methylated ZDHHC1 multi-target stool DNA panel, fecal hemoglobin and a 109-taxon random-forest signature, with the fecal immunochemical test (FIT) as comparator and its false-positive rate rising from 2.4% to 9.4% when combined with microbiome features; (d) autoimmune disease - Prevotella copri at species level in rheumatoid arthritis and pre-symptomatic rheumatoid arthritis, distinguished from genus-level Prevotella spp. in systemic lupus erythematosus, together with depleted Eubacterium rectale, the core rheumatoid-arthritis biobank species, a fecal cytokine and gut-barrier panel and the 10/9-species inflammatory bowel disease

diagnostic panels. (B) Six-step fecal SCFA quantification pipeline: stool collection with standardized storage at -80 C immediately after collection, direct extraction from stool, optional derivatization (isobutyl chloroformate or BSTFA, omitted in derivatization-free protocols), instrumental analysis by GC-MS as the reference method or by GC-FID or LC-MS/MS with isotope-labeled internal standards, the quantified profile of acetate, propionate, butyrate and the branched-chain SCFAs iso-butyrate and iso-valerate, and integration as Host Feature MLP input alongside taxonomic and dietary embeddings. Cited in Sections 3.1.5 and 4.1; see Table 2 for the full disease list.

Disease-specific extended biomarker panels for T1DM, MOS/PCOS, colorectal cancer and autoimmune disease, alongside the six-step fecal SCFA quantification pipeline from stool collection to model input. Cited in Sections 3.1.5 and 4.1.
